# Impact of prior outpatient antibiotic use on mortality for community acquired pneumonia: a retrospective cohort study

**DOI:** 10.1186/1756-0500-1-120

**Published:** 2008-12-01

**Authors:** Eric M Mortensen, Marcos I Restrepo, Jacqueline A Pugh, Antonio Anzueto

**Affiliations:** 1 VERDICT Research Program, Audie L Murphy VA Hospital, 7400 Merton Minter Blvd (11C6), San Antonio, Texas, 78229, USA; 2 Divisions of General Medicine/Hospital Medicine, the University of Texas Health Science Center at San Antonio, 7703 Floyd Curl Drive, San Antonio, Texas, 78229, USA; 3 Division of Pulmonary and Critical Care Medicine, the University of Texas Health Science Center at San Antonio, 7703 Floyd Curl Drive, San Antonio, Texas, 78229, USA

## Abstract

**Background:**

The purpose of this study was to examine whether prior outpatient antibiotic use is associated with increased 30-day mortality, after adjusting for potential confounders, for those subsequently hospitalized with pneumonia.

**Methods:**

A retrospective cohort study conducted at two tertiary teaching hospitals. Eligible subjects were admitted with a diagnosis of, and had a chest x-ray consistent with, community-acquired pneumonia. Our primary analysis was a multivariable logistic regression with the dependent variable of 30-day mortality.

**Results:**

Data was abstracted on 733 subjects at the two hospitals. Mortality was 8.1% at 30-days. At presentation, 55% of subjects were low risk, 33% were moderate risk, and 12% were high risk. In our cohort 17% (n = 128) of subjects received antibiotics within 30-days of presentation. Unadjusted mortality for those who had received prior antibiotics was 7.0% vs. 8.3% for those who had not (p = 0.6). In the multivariable analysis prior use of antibiotics (odds ratio 0.98, 95% confidence interval 0.5–2.1) was not significantly associated with 30-day mortality.

**Conclusion:**

Receipt of prior outpatient antibiotics is not significantly associated with 30-day mortality for patients hospitalized with pneumonia. Our study supports current efforts to increase the number of patients with pneumonia who are treated as outpatients.

## Background

Pneumonia, along with influenza, is the seventh leading cause of death and the leading cause of infectious death in the United States [1]. Although mortality due to pneumonia decreased significantly with the introduction of antibiotics in the 1950s, since that time mortality has been stable or increasing [2]. Recently substantial attention has been focused on increasing the percentage of patients safely treated for community-acquired pneumonia as outpatients rather than requiring initial hospitalization [3]. However, up to 20% of patients fail outpatient treatment and require subsequent hospitalization for pneumonia [4]. There have been few studies that have examined whether receiving prior outpatient antibiotic treatment for pneumonia is associated with worse outcomes [5-7], and the results are conflicting.

The study aim was to examine whether prior outpatient antibiotic use is associated with increased 30-day mortality, after adjusting for potential confounders and severity of illness at presentation, for patients hospitalized with community-acquired pneumonia.

## Methods

This a retrospective cohort study of patients hospitalized with pneumonia at 2 academic tertiary care hospitals in San Antonio, Texas. Both hospitals are teaching affiliates of the University of Texas Health Science Center at San Antonio. The Institutional Review Board of the University Health Science Center at San Antonio approved the research protocol with exempt status.

### Study Sites/Inclusion and Exclusion Criteria

We identified all patients admitted to the study hospitals between January 1, 1999 and December 1, 2002 with a primary discharge diagnosis of pneumonia (ICD-9 codes 480.0–483.99 or 485–487.0) or secondary discharge diagnosis of pneumonia with a primary diagnosis of respiratory failure (518.81) or sepsis (038.xx). Subjects were included if they were 1) greater than 18 years of age, 2) had an admission diagnosis of pneumonia, and 3) had a radiographically confirmed infiltrate or other finding consistent with pneumonia on chest x-ray or CT obtained within 24 hours of admission.

Exclusion criteria included 1) having been discharged from an acute care facility within 14 days of admission, 2) transfer after being admitted to another acute care hospital, 3) being a resident of a skilled nursing facility prior to admission, and 4) being comfort measures only on this admission. If a subject was admitted more than once during the study period, only the first hospitalization was abstracted.

### Data Abstraction

Chart review data included: demographics, comorbid conditions, physical examination findings, laboratory data, and chest radiograph reports. In addition, data on important processes of care measures for patients hospitalized with pneumonia were also abstracted: time to first dose of antibiotics, collection of blood cultures prior to antibiotic administration, and obtaining blood cultures and oxygen saturation measurement within 24 hours of presentation [8]. Antimicrobial therapy was considered guideline-concordant if it agreed with either the 2000 Infectious Diseases Society of America or 2001 American Thoracic Society guidelines [9,10], which are similar to the recommendations from the 2007 joint guidelines from these societies [11]. Information on all outpatient medications that were either 1) reported as currently being taken by the patient at presentation, or 2) listed in the electronic medical record, were recorded. Patients were defined as having prior antibiotic use if they had received a prescription within the 30-days prior to hospital presentation.

### Diagnostic criteria

Microbiologic data results were reviewed, and a microbiologic cause was assigned independently by two of the investigators (MIR and EMM). The cause of pneumonia was stratified as definitive or presumptive. The diagnosis was considered definitive if one of the following conditions were met: (1) positive blood cultures for bacterial or fungal pathogens were obtained (in the absence of extra-pulmonary source of infection); (2) pleural fluid cultures yielded a bacterial pathogen; (3) endotracheal aspirates with moderate or heavy growth of bacterial pathogens were observed; (4) significant quantitative culture growth from bronchoscopic respiratory samples were observed (protected specimen brush cultures of at least 10^3 ^cfu/mL, and in bronchoalveolar lavage of at least 10^4 ^cfu/mL). A presumptive diagnosis was made if a qualitative valid sputum sample yielded one or more predominant bacterial pathogens. Definitive and presumptive causes were combined for reporting purposes. When two or more microbiologic causes were present, the patient was considered to have a polymicrobial infection. A patient was considered to have pneumonia of unknown cause if microbiologic studies were not performed or were inconclusive.

### Risk Adjustment

The pneumonia severity index was used to assess severity of illness at presentation [4]. The pneumonia severity index is a validated prediction rule for 30-day mortality in patients with community-acquired pneumonia. This rule is based on three demographic characteristics, five comorbid illnesses, five physical examination findings, and seven laboratory and radiographic findings from the time of presentation. Patients are classified into five risk classes with 30-day mortality ranging from 0.1% for class I to 27% for class V for patients enrolled in the PORT cohort study [4].

### Outcome

We used 30-day mortality as the outcome for this study. Previous research has demonstrated that 30-day mortality is primarily due to the pneumonia rather than other co-existing co-morbid conditions.[12,13] Mortality was assessed using information from the Texas Department of Health and Department of Veteran Affairs clinical database. Mortality status was assessed through December 2002.

### Statistical Analyses

In a post-hoc power calculation, assuming an alpha of 0.05 and beta of 0.2, we were able to detect a 1.7× difference in mortality between the 2 groups.

Univariate statistics were used to test the association of sociodemographic and clinical characteristics with all-cause 30-day mortality. Categorical variables were analyzed using the Chi-square test and continuous variables were analyzed using Student's t-test. A multivariable logistic regression model was derived with 30-day mortality as the dependent variable, and the pneumonia severity index, process of care measures (initial antibiotics within 8 hours and whether antimicrobial therapy was guideline concordant), and prior receipt of antibiotics within the 30-days prior to presentation as independent variables. All analyses were performed using STATA version 9 (Stata Corporation, College Station, Texas).

## Results

Data was abstracted on 733 patients at the two hospitals. The mean age was 59 years with a standard deviation of 16 years. The population was 78% male, 84% were admitted through the emergency department, and 20% were admitted to the intensive care unit (ICU) within the first 24 hours after admission. Mortality was 8.1% at 30-days and 12.1% at 90-days. By pneumonia severity index, 55% were low risk (pneumonia severity index classes I-III), 33% were moderate risk (pneumonia severity index class IV), and 12% were high risk (pneumonia severity index class V).

In our cohort 17% (n = 128) of subjects received antibiotics within 30-days of presentation. Unadjusted mortality for those who had received prior antibiotics was 7.0% vs. 8.3% for those who had not (p = 0.6). There was also no significant difference in length of stay (prior antibiotics- 6.9 days with a standard deviation (SD) of 8.7 vs. 7.8 days and a SD of 16.7, p = 0.5) or rate of ICU admission (14% vs. 20%, p = 0.1). Table 1 shows the demographic factors and clinical characteristics for this population by prior receipt of antibiotics. Table 2 shows the most commonly received antibiotics prior to admission. These most commonly used antibiotics, in descending order, were levofloxacin (n = 31), amoxicillin (n = 17), azithromycin (n = 16), amoxicillin-clavulanate (n = 16), ciprofloxacin (n = 7), clarithromycin (n = 6), and erythromycin (n = 5).

**Table 1:** Subject demographic and clinical characteristics by receipt of antibiotics prior to admission*

	**Receipt of Antibiotics Prior to Admission**		
**Variable**	**Yes (n = 128)**	**No (n = 605)**	**p-value**
Age, years (standard deviation)	58.2 (16.1)	59.7 (16.1)	0.3
Men	90 (70)	482 (86)	0.02
Admitted through emergency department	104 (81)	509 (89)	0.4
Admitted to intensive care within 24 hours	18 (14)	125 (20)	0.09
***Preexisting Comorbid Conditions***			
Congestive heart failure	16 (13)	90 (15)	0.5
Chronic pulmonary disease	35 (27)	16 (22)	0.9
History of stroke	10 (8)	72 (12)	0.2
Chronic liver disease	3 (2)	34 (6)	0.1
History of malignancy	12 (9)	59 (10)	0.9
Renal insufficiency	13 (10)	58 (10)	0.8
***History, Physical, Laboratory, and Radiographic Data***			
Altered mental status	12 (9)	56 (9)	0.9
Respiratory rate > 30 per minute	10 (8)	64 (11)	0.3
Systolic blood pressure < 90 mmHg	2 (2)	15 (2)	0.5
Heart rate > 125 per minute	12 (9)	86 (14)	0.1
Temperature < 95° or > 104°	0 (0)	20 (3)	0.04
Arterial pH < 7.35	5 (4)	4 (7)	0.2
Arterial oxygenation saturation < 90%	28 (22)	137 (23)	0.9
Hematocrit < 30%	9 (7)	56 (9)	0.4
Serum blood urea nitrogen > 30 mg/dL	27 (21)	120 (20)	0.8
Serum glucose > 250 mg/dL	14 (11)	60 (10)	0.7
Serum sodium < 130 meq/L	23 (18)	88 (15)	0.3
Pleural effusion on chest radiograph	27 (21)	147 (24)	0.4
***Pneumonia Severity Index***			
Class I-III	74 (58)	330 (54)	
Class IV	45 (35)	198 (33)	
Class V	9 (7)	77 (13)	0.2

* Data are presented as number (%) or mean (standard deviation)

**Table 2:** Antibiotics received within 30-days of hospital presentation (n = 128)

**Antibiotic**	**N**
Amoxicillin	17
Amoxicillin-clavulanate	16
Azithromycin	16
Cefuroxime axetil	4
Ciprofloxacin	7
Clarithromycin	6
Doxycycline	2
Erythromycin	5
Gatifloxacin	3
Levofloxacin	31
Other	21

Regarding organisms isolated there were significantly less organisms isolated in those with prior antibiotics with 15% versus 26% for those who did not receive antibiotics prior to admission (p = 0.006). In addition, there was a trend towards lower bacteremia in those who had received outpatient antibiotics (9% vs. 16%, p = 0.07). Table 3 shows the most commonly isolated organisms. The only isolated organisms that were significantly different between groups were *Streptococcus pneumoniae *(2% in the prior antibiotic group versus 10% in the non-use group) and *Haemophilus influenza*e (0% vs 3% respectively). There were no significant differences in resistance for either *Streptococcus pneumoniae *(50% penicillin sensitive for prior antibiotic users vs. 48% for non-users, p = 0.9) or *Staphylococcus aureus *(50% methicillin sensitive for prior antibiotic users vs. 82% for non users, p = 0.1).

**Table 3:** Etiologic diagnosis by receipt of antibiotics prior to admission versus non-receipt*

**Microorganisms**	**Prior Antibiotic Use n = 128 N (%)**	**No Prior Antibiotic Use n = 605 N (%)**	**P-value**
*Streptococcus pneumoniae*	2 (2)	58 (10)	0.003
*Staphylococcus aureus*	4 (3)	34 (6)	0.2
*Pseudomonas aeruginosa*	6 (5)	14 (2)	0.1
*Haemophilus influenzae*	0 (0)	19 (3)	0.04
*Klebsiella pneumoniae*	1 (1)	7 (1)	0.8
*Escherichia coli*	2 (1)	7 (1)	0.7
*Proteus mirabilis*	0 (0)	3 (1)	0.4
Miscellaneous **	2 (2)	8 (1)	0.6
Polymicrobial	2 (2)	10 (2)	0.9
No pathogen isolated	109 (85)	445 (74)	0.006

* Percentages have been rounded and may not sum 100.

** Miscellaneous consists of *Acinetobacter *spp., *Aspergillus *spp., *Haemophilus parainfluenzae, Enterococcus *spp. and *Streptococcus *spp.

In the multivariable analysis (Table 4), after adjusting for potential confounders, prior use of antibiotics (odds ratio 0.98, 95% confidence interval 0.5–2.1) was not significantly associated with 30-day mortality.

**Table 4:** Results of the multivariable logistic regression model with 30-day mortality as the dependent variable

**Variable**	**Odds Ratio**	**95% Confidence Interval**
PSI class	2.0	1.5–2.6
Initial antibiotics within 8 hours of admission	1.2	0.7–2.1
Use of guideline concordant antibiotics	0.8	0.4–1.4
Antibiotic use prior to admission	0.98	0.5–2.1

## Discussion

We found that outpatient use of antibiotics prior to initial hospital presentation was not significantly associated with 30-day mortality for subjects hospitalized with pneumonia. In addition, we found no significant differences in other important clinical variables or outcomes, such as pneumonia severity index class, length of stay, and rates of ICU admission, between those who did, and did not, receive antibiotics prior to admission. These results are reassuring that prior antibiotic therapy is not associated with increased mortality and support the current measures to increase the number of patients with community-acquired pneumonia that are treated as outpatients.

The prior literature regarding the association of prior antibiotic therapy and mortality is mixed. Our study supports the findings of the recent study by van de Garde et al. [5] of 1090 patients in the Netherlands who found that prior antibiotic use was not associated with increased in-hospital mortality (OR 1.09, 95% CI 0.65–1.83). In addition, Johnson et al[7] found in a study utilizing administrative data of over 21000 subjects hospitalized with pneumonia in Alberta, Canada that after risk adjustment receipt of any antibiotic prior to admission was associated with decreased mortality (OR 0.66, p < 0.0001). However, our findings contrast with that of Meehan et al. [6,8], a nationwide study based on Medicare quality improvement data, who found a small increase in mortality for those who received antibiotics prior to admission (unadjusted mortality 17.6% vs. 14.4%, multivariable model OR 1.32, 95% CI 1.28–1.37.) It is unclear however if this difference was due to a direct impact of prior antibiotic therapy, or was actually a marker for other, unmeasured, parts of the medical care process, as was demonstrated by the protective association of blood cultures prior to antibiotics in this same paper [8]. Also, our study may have had insufficient sample size to detect this small of a mortality difference. With our current sample we would have 67% power to detect a similar difference in mortality.

As one would expect those who received prior antibiotic therapy had significantly lower rates of bacterial identification with lower rates of isolation of *Streptococcus pneumoniae *and *Haemophilus influenzae*. Interestingly we saw no significant difference in rates of penicillin resistance for *Streptococcus pneumoniae *or methicillin resistance *Staphylococcus aureus *by prior antibiotic use. However, this may be due to the small number of subjects who had sputum cultures obtained (47%) or blood cultures obtained prior to IV antibiotics (76%).

Although our study was retrospective and subject to the recognized limitations of such studies, we carefully assembled our cohort from complete patient discharge data to avoid ascertainment bias. Additionally, during chart abstraction we encountered a very small amount (<5%) of missing data. Our sample was predominantly men due to the inclusion of a VA hospital and it is possible, but unlikely, that women may have differential responsiveness to antibiotic use as compared to men. Finally, we are unable to ensure that all prior outpatient antibiotics were prescribed for pneumonia and not another indication. However, we do not believe that more than a very small number of patients would have had another bacterial infection (e.g., cellulitis, urinary tract infection) so closely to the episode of pneumonia. This is also supported by the fact that almost 1/3 of the antibiotics given were respiratory fluoroquinolones, which during this time period, and at these institutions, were the preferred outpatient therapy for CAP patients with risk factors for drug resistant *Streptococcus pneumoniae*. This supports our contention that the vast majority of these antibiotics were used for previously diagnosed pneumonia.

## Conclusion

We found no association with prior outpatient use of antibiotics within 30-days of hospital presentation and mortality for subjects hospitalized with community-acquired pneumonia. In addition, we found no difference in other markers for increased severity by prior antibiotic use including length of stay, severity of illness at presentation, and rate of ICU admission. Further research is needed to determine if patients with community-acquired pneumonia, who have had recent antibiotic use, are at risk for multi-drug resistant organisms, which requires much broader antimicrobial therapy.

## Abbreviations

CI: Confidence Interval; cfu: colony forming units; CT: Computed axial Tomography; ICD-9: International statistical Classification of Diseases and related health problems; version 9; ICU: Intensive Care Unit; mL: milliliter; n: Number; OR: Odds Ratio; PORT: Patient Outcomes Research Team.

## Authors' contributions

EMM originated and coordinated the study, obtained funding, contributed to the analysis of the data, and contributed to preparation of the paper. MIR, AA, and JP contributed to the design of the study, the analysis of the data, and preparation of the paper. All authors read and approved the final manuscript.

## References

[B1] Hoyert DL, Arias E, Smith BL (2001). Deaths: Final Data for 1999. Natl Vital Statistics Report.

[B2] Gilbert K, Fine MJ (1994). Assessing prognosis and predicting patient outcomes in community-acquired pneumonia. Seminars in Respiratory Infections.

[B3] Atlas SJ, Benzer TI, Borowsky LH, Chang Y, Burnham DC, Metlay JP, Halm EA, Singer DE (1998). Safely increasing the proportion of patients with community-acquired pneumonia treated as outpatients: an interventional trial. Archives of Internal Medicine.

[B4] Fine MJ, Auble TE, Yealy DM, Hanusa BH, Weissfeld LA, Singer DE, Coley CM, Marrie TJ, Kapoor WN (1997). A prediction rule to identify low-risk patients with community-acquired pneumonia. N Engl J Med.

[B5] Garde EM van de, Souverein PC, Bosch JM van den, Deneer VH, Goettsch WG, Leufkens HG (2006). Prior outpatient antibacterial therapy as prognostic factor for mortality in hospitalized pneumonia patients. Respir Med.

[B6] Meehan TP (1998). Quality of care for elderly patients with pneumonia. In Reply. JAMA.

[B7] Johnson D, Carriere KC, Jin Y, Marrie T (2004). Appropriate antibiotic utilization in seniors prior to hospitalization for community-acquired pneumonia is associated with decreased in-hospital mortality. J Clin Pharm Ther.

[B8] Meehan TP, Fine MJ, Krumholz HM, Scinto JD, Galusha DH, Mockalis JT, Weber GF, Petrillo MK, Houck PM, Fine JM (1997). Quality of care, process, and outcomes in elderly patients with pneumonia. JAMA.

[B9] Niederman MS, Mandell LA, Anzueto A, Bass JB, Broughton WA, Campbell GD, Dean N, File T, Fine MJ, Gross PA (2001). Guidelines for the management of adults with community-acquired pneumonia. Diagnosis, assessment of severity, antimicrobial therapy, and prevention. Am J Respir Crit Care Med.

[B10] Bartlett JG, Dowell SF, Mandell LA, File TM, Musher DM, Fine MJ (2000). Practice guidelines for the management of community-acquired pneumonia in adults. Infectious Diseases Society of America. Clin Infect Dis.

[B11] Mandell LA, Wunderink RG, Anzueto A, Bartlett JG, Campbell GD, Dean NC, Dowell SF, File TM, Musher DM, Niederman MS (2007). Infectious diseases society of america/american thoracic society consensus guidelines on the management of community-acquired pneumonia in adults. Clin Infect Dis.

[B12] Mortensen EM, Kapoor WN, Chang CC, Fine MJ (2003). Assessment of mortality after long-term follow-up of patients with community-acquired pneumonia. Clin Infect Dis.

[B13] Mortensen EM, Coley CM, Singer DE, Marrie TJ, Obrosky DS, Kapoor WN, Fine MJ (2002). Causes of death for patients with community-acquired pneumonia: results from the Pneumonia Patient Outcomes Research Team cohort study. Arch Intern Med.

